# Long noncoding RNA KCNMB2-AS1 acts as an oncogene in ovarian cancer

**DOI:** 10.3724/abbs.2023228

**Published:** 2023-09-14

**Authors:** Siyu Cao, Min Liu, Ziyi Guo, Yanli Li

**Affiliations:** 1 Department of Gynecologic Oncology Fudan University Shanghai Cancer Center Shanghai 200032 China; 2 Lab for Noncoding RNA and Cancer School of Life Sciences Shanghai University Shanghai 200444 China

Ovarian cancer (OC), a gynecologic cancer, has a poor prognosis and limited treatment options. More than 75% of patients are initially diagnosed with advanced OC [1]. The 5-year overall survival rate of OC has remained virtually unchanged, which means it is still low [2]. Therefore, research on OC is unceasing, and new therapeutic targets and biomarkers are urgently needed [3].

Noncoding RNAs (ncRNAs) have been demonstrated to be important regulators of multiple diseases, including cancer, leukemia, cardiovascular disease and neurological disorders [4]. LncRNA is a type of ncRNA that contains more than 200 nt, lacks a meaningful open reading frame (ORF) and does not have protein-coding function [5]. LncRNAs also play a very important role in OC regulation [6]. In recent years, KCNMB2-AS1 has been studied in other diseases, such as bladder cancer [7] and esophageal cancer [8], but its function in OC is still unclear.

First, the relative expression of KCNMB2-AS1 was determined in 8 normal tissues (NT) and 6 OC patient tissues ( Figure 1A). The expression of KCNMB2-AS1 was also found to be lower in normal ovarian epithelial cell lines than that in OC cell lines ( Supplementary Figure S1A). Both results suggested that KCNMB2-AS1 was highly expressed in OC. The results of nucleocytoplasmic separation experiments indicated that KCNMB2-AS1 was more predominant in the cytoplasm ( Supplementary Figure S1B–D).

**Figure 1:**
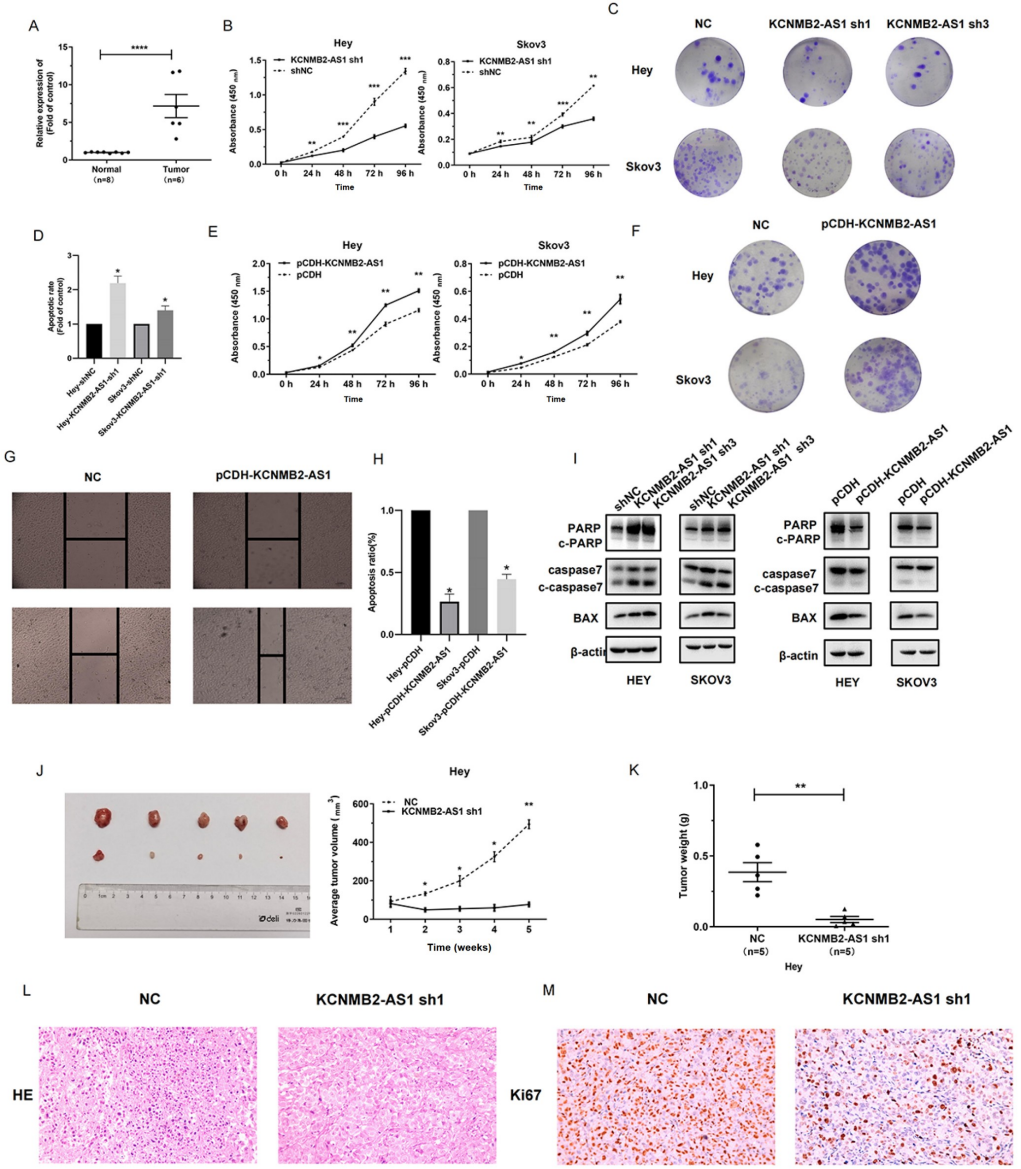
Figure 1 KCNMB2-AS1 acts as oncogene *in vitro* and *in* *vivo* (A) Relative expression of KCNMB2-AS1 in patient tissues. (B) Cell Counting Kit-8 (CCK-8) assay of Hey and Skov3 cells with KCNMB2-AS1 knockdown. (C) Representative images of colony formation of OC cells by staining with crystal violet after knockdown of KCNMB2-AS1. (D) Proportions of apoptotic Hey and Skov3 cells with KCNMB2-AS1 knockdown. (E) CCK-8 assay of Hey and Skov3 cells with KCNMB2-AS1 upregulation. (F) Representative images of colony formation of OC cells by staining with crystal violet after upregulation of KCNMB2-AS1. (G) Wound healing assay of Hey and Skov3 cells. (H) Proportions of apoptotic Hey and Skov3 cells with KCNMB2-AS1 upregulation. (I) Protein levels of apoptosis markers in OC cells with KCNMB2-AS1 knockdown and upregulation. (J) Xenograft animal model showing the volume of subcutaneous tumors with KCNMB2-AS1 knockdown ( n=5 per group). (K) Weight of subcutaneous tumors. (L) Alterations in HE staining in xenograft tumors were investigated by IHC staining. (M) Alterations in Ki67 expression in xenograft tumors were investigated by IHC staining. Data are presented as the mean±SD of three independent experiments. * P<0.05, ** P<0.01, and *** P<0.001.

To further ascertain the regulatory role of KCNMB2-AS1 in OC, KCNMB2-AS1 was knocked down in two OC cell lines, Hey and Skov3 ( Supplementary Figure S1E). In both cell lines, proliferation was markedly reduced in cells with KCNMB2-AS1 knockdown ( Figure 1B and Supplementary Figure S1F). The results of the colony formation assay verified this conclusion ( Figure 1C and Supplementary Figure S1G). After knockdown of KCNMB2-AS1, there was also a significant increase in the percentage of apoptotic cells ( Figure 1D and Supplementary Figure S1H).

To confirm the function of KCNMB2-AS1, the expression of KCNMB2-AS1 was upregulated in the cell lines ( Supplementary Figure S1J). Through the CCK-8 assay, it was confirmed that upregulation of KCNMB2-AS1 led to increased cell proliferation ( Figure 1E). The same conclusion was reached in the colony formation assay ( Figure 1F and Supplementary Figure S1K). The results of the wound healing assay revealed that the migratory ability of OC cells was significantly increased ( Figure 1G and Supplementary Figure S1L). Moreover, the proportion of apoptotic cells was significantly decreased after upregulation of KCNMB2-AS1 ( Figure 1H and Supplementary Figure S1M). These results provide preliminary evidence that KCNMB2-AS1 exerts a cancer-promoting effect. Then, western blot analysis was used to measure the expressions of apoptosis-associated markers. The results showed that KCNMB2-AS1 knockdown increased the expression of Bax and decreased the expressions of caspase-7 and PARP ( Figure 1I) and that KCNMB2-AS1 overexpression decreased the expression of Bax and increased the expressions of caspase-7 and PARP ( Figure 1I).

Subsequently, animal experiments were performed to further validate the cancer-promoting effect of KCNMB2-AS1. To verify the *in vitro* results, xenograft tumor models were established by subcutaneous injection of Hey cells transduced with empty vector or a lentiviral plasmid containing a shRNA targeting KCNMB2-AS1 (sh-KCNMB2-AS1 sense: 5′-CCGGGCTAGCTTCTCGTGTGCTTGTCTCGAGACAAGCACACGAGAAGCTAGCTTTTTG-3′, and antisense: 5′-AATTCAAAAAGCTAGCTTCTCGTGTGCTTGTCTCGAGACAAGCACACGAGAAGCTAGC-3′). The volumes of the tumors formed by sh-KCNMB2-AS1 Hey cells were significantly smaller than those of the tumors in the negative control (NC) group. When KCNMB2-AS1 was knocked down, the tumor growth rate decreased significantly. The tumor volume in the knockdown group was also apparently lower than that in the control group ( Figure 1J). In addition, the tumor volume growth curve of the KCNMB2-AS1 knockdown group was significantly flatter than that of the control group ( Figure 1J). Hence, knockdown of KCNMB2-AS1 decreased OC cell proliferation. The tumor weights also confirmed the above conclusion ( Figure 1K). The results of IHC also confirmed the decrease in the tumorigenic ability of Hey cells after knockdown of KCNMB2-AS1 ( Figure 1M,N). The FISH of patient samples and normal samples also confirmed that KCNMB2-AS had higher expression in EOCtissues ( Figure 2).

**Figure 2:**
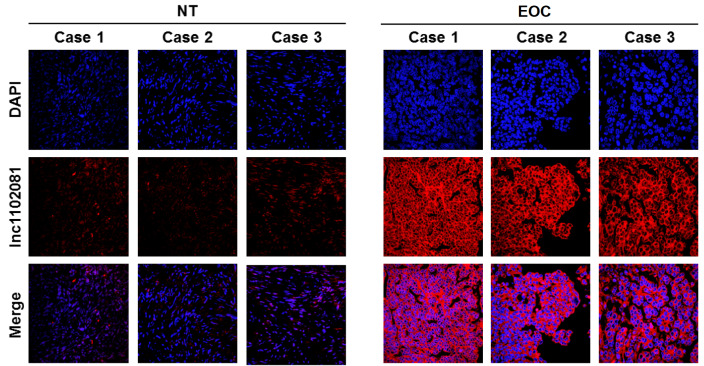
Figure 2 Subcellular localization of KCNMB2-AS1 in 3 NT and 3 EOC tissues detected by FISH It had higher expression in EOC tissues. Blue: DAPI. Red: KCNMB2-AS1.

In summary, KCNMB2-AS1 was found to play a cancer-promoting role in OC. First, KCNMB2-AS1 was highly expressed in patient samples and cell lines. We demonstrated that KCNMB2-AS1 promoted OC cell proliferation and migration and reduced their apoptosis. In support of the results obtained *in vitro*, knockdown of KCNMB2-AS1 significantly reduced tumor volume and weight *in vivo*, as shown in Figure 1I. KCNMB2-AS1 was illustrated to promote OC. Our results demonstrate the regulatory ability of KCNMB2-AS1 in OC, further indicating its ability to be considered a potential therapeutic target.

## Supporting information

Supplementary

## Supplementary Data

Supplementary data is available at *Acta Biochimica et Biophysica Sinica* online.
