# Supplementary material for: Long noncoding RNA KCNMB2-AS1 acts as an oncogene in ovarian cancer: KCNMB2-AS1 acts as an oncogene in ovarian cancer
Source: Acta Biochim Biophys Sin (Shanghai). 2023 Sep 14;55(11):1844–6. doi: 10.3724/abbs.2023228 (PMC10679871; doi:10.3724/abbs.2023228)
Supplement: Supplementary [file Supplementary.pdf]

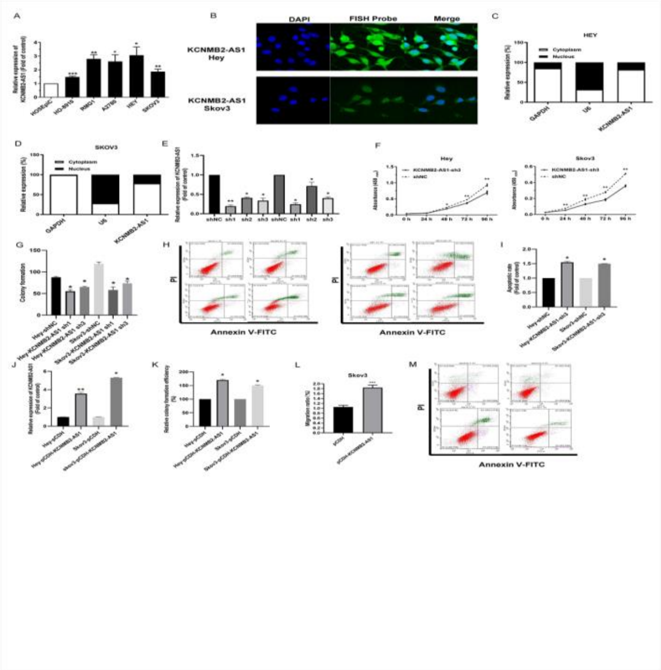

Supplementary Figure S1. Roles of KCNMB2-AS1 in OC cells (A) Expression of KCNMB2-AS1 in normal ovarian epithelial cell lines and OC cell lines. (B) Fluorescence *in situ* hybridization (FISH) assay showing the distribution of KCNMB2-AS1 in OC cells. (C) Nucleocytoplasmic separation assay showing the distribution of KCNMB2-AS1 in Hey cells. (D) Nucleocytoplasmic separation assay showing the distribution of KCNMB2-AS1 in Skov3 cells. (E) qRT-PCR analysis was performed to confirm the expression of KCNMB2-AS1 in both Hey and Skov3 cells transduced with shRNA targeting KCNMB2-AS1. (F) Cell Counting Kit-8 (CCK-8) assay of Hey and Skov3 cells with KCNMB2-AS1 knockdown. (G) Statistical analysis of the colony formation assay data. (H) Flow cytometric apoptosis analysis of KCNMB2-AS1 knockdown cells. (I) Proportion of apoptotic Hey and Skov3 cells with KCNMB2-AS1 knockdown. (J) The expression of KCNMB2-AS1 in both Hey and Skov3 cells after treatment with the KCNMB2-AS1 overexpression vector. (K) Statistical analysis of the colony formation assay data. (L) Quantitative analysis of the wound healing assay data. (M) Flow cytometric apoptosis analysis of KCNMB2-AS1 upregulation. Data are presented as the mean ± SD of three independent experiments. *P< 0.05, **P< 0.01, and ***P< 0.001.
